# Heat Shock Differentially Compromises Embryonic Development and Gene Expression in a Mouse Embryoid Body Model System

**DOI:** 10.3390/ani15223293

**Published:** 2025-11-14

**Authors:** Payungsuk Intawicha, Kamonthip Sonsiri, Chun-Ru Yang, Neng-Wen Lo, Pin-Chi Tang, Jyh-Cherng Ju

**Affiliations:** 1 Department of Animal Science, National Chung Hsing University, Taichung 40227, Taiwan; payungsuk.in@up.ac.th (P.I.); d109037006@mail.nchu.edu.tw (K.S.); littlebaby37@yaoo.com.tw (C.-R.Y.); pctang@dragon.nchu.edu.tw (P.-C.T.); 2 School of Agriculture and Natural Resources, University of Phayao, Phayao 56000, Thailand; 3 Department of Animal Science and Biotechnology, Tunghai University, Taichung 407224, Taiwan; nlo@thu.edu.tw; 4 Graduate Institute of Biomedical Sciences, China Medical University & Hospital, Taichung 404333, Taiwan

**Keywords:** embryoid body, embryonic stem cells, germ layers, heat shock, mouse

## Abstract

This study established an in vitro model to investigate peri- and post-implantation embryo development under heat shock conditions. In Experiment 1, we compared the gene expression profiles of the three germ layers in in vivo mouse embryos with those of mEBs derived from mouse embryonic stem cells. mEBs (Days 1–5) closely resembled developing E5.5–E6.5 embryos, expressing key germ layer markers: *nestin* (ectoderm), *flk-1* (mesoderm), and *ttr* (endoderm). In Experiment 2, mEBs were randomly allocated to one of the following treatments: a control group (37 °C) or a heat shock group (39 °C or 41 °C) for 12 h or 24 h. Timing of germ layer marker expression, including *ttr*, was delayed by 1–2 days in the heat-shocked groups compared with the control group. Morphological analysis of mEBs revealed that the peripheral cell layer exhibited signs of disassembly or degeneration and became increasingly apoptotic under heat shock conditions. Additionally, these mEBs showed increased expression of heat shock proteins 70 and 72 in response to elevated temperatures and prolonged heat shock durations. Mouse EBs closely mimic in vivo-developing embryos in their germ layer marker expression profiles. In vitro heat shock delays germ layer marker gene expression and induces heat shock protein 72 expression in mEBs.

## Simple Summary

High environmental temperatures can negatively affect embryo development in animals. In this study, we used mouse embryoid bodies (mEBs) grown from embryonic stem cells to model early embryo development in the laboratory. Mouse EBs developed in a way similar to real mouse embryos during the peri- and post-implantation stages. When exposed to heat shock (39 °C or 41 °C), mEBs showed delayed expression of key genes for the three germ layers and increased cell death. Heat shock also triggered the production of heat shock proteins, which help protect cells from heat stress. Mouse EBs can be used to study how heat stress affects early embryo development and may help improve animal reproduction under high-temperature conditions.

## 1. Introduction

Heat stress significantly impacts livestock production in tropical and subtropical regions, particularly during the long-day period [1]. In most domestic animals, including rabbits [2], pigs [3], and cattle [4], heat stress has been reported to compromise nutritional, physiological, and reproductive functions [5]. It has a detrimental effect on testicular functions, such as inhibiting spermatogenesis in pigs [6] and cows [7]. An *in vivo* study showed that the conception rate in dairy cows was reduced following artificial insemination with sexed semen performed in summer [8]. Heat stress affects the cellular and molecular mechanisms underlying ovarian, granulosa cell, and uterine functions [9], and it leads to a decline in reproductive hormones, oocyte quality, fertilization, and embryo development [10]. Research into the effects of heat stress on preimplantation embryos has garnered considerable attention because of its direct impact on the cellular and molecular processes governing embryonic growth and differentiation [11]. However, conducting *in vivo* studies on the effects of heat stress during embryogenesis is often restricted by ethical and sampling issues related to animal use, as well as the technical challenges of acquiring embryos at specific developmental stages.

Oocytes at the germinal vesicle stage (i.e., cumulus–oocyte complexes) exposed to in vitro heat shock from 40 °C to 41 °C exhibit increased abnormalities in nuclear and cytoplasmic maturation [12]. Heat stress also alters oocyte and cumulus cell functions, including mitochondrial oxidative phosphorylation, spindle formation, cytoskeleton components, maternal transcription, and matrix metallopeptidase 9, as well as decreasing progesterone production. This, in turn, affects oocyte protein synthesis, disrupts oocyte maturation, and compromises subsequent development [13]. In other words, heat stress can induce oxidative stress, leading to DNA damage through the elevation of reactive oxygen species, apoptosis, and lipid peroxidation, which result in abnormal gene and protein expression in bovine preimplantation embryos produced *in vitro* [14]. Therefore, prolonged exposure to high temperatures frequently causes oxidative damage to essential biological components, such as lipids, proteins, and DNA, ultimately inducing germ cell death [15].

In embryonic development, heat stress has been found to significantly impact early embryogenesis, notably disrupting the intricate processes of oocyte maturation, cellular differentiation, and overall development [16]. Increased temperatures can alter DNA structures and its gene expressions, and in turn hinder the formation of the three germ layers, i.e., ectoderm, mesoderm, and endoderm, during early development. This procedure is essential for the later development of embryonic tissues and organs. [17]. Consequently, heat stress has been shown to considerably influence oocyte maturation, cellular differentiation, and overall embryonic development, resulting in impaired developmental advancement [18]. The adverse effects of high ambient temperatures and experimentally induced heat stress on fetal and placental growth have been frequently reported in humans, ruminants, and murine species [19].

Environmental thermal stresses, either acute or chronic elevated temperatures, have received more attention because they have been recognized as significant determinants that affect early embryonic development and eventual animal health. It is likely that elevated ambient temperatures jeopardize mammalian reproduction by disrupting the intricate processes of peri- or post-implantation embryogenesis via germ layer differentiation [20]. In avian species, it has been reported that heat stress affects the complex process of germ layer differentiation, with it also being recognized as a teratogenic agent that impairs normal embryonic development in various animal species, including cattle [21], chickens [22] and mice [23]. Increased temperature hinders germ layer development and organogenesis, frequently resulting in congenital abnormalities or embryonic loss [24]. Even short hyperthermic exposure during early gestation diminishes essential transcription factors like Oct4 in blastocysts and embryonic stem cells, leading to decreased cleavage and blastocyst formation rates [23]. To the best of our knowledge, most of the research has been focused on oocyte competence or cleavage-stage embryos, with limited investigation into the effects of heat on germ layer lineage specification in mammals, possibly due to its difficulty to study. To address these gaps, it is critical to understand how early thermal stress disrupts developmental programming and compromises offspring viability.

Heat shock proteins (HSPs) play an essential role in the cellular stress response by acting as molecular chaperones, particularly in protecting cells from injuries caused by elevated temperatures [25]. HSPs play significant roles in modulating the immune response, regulating apoptotic processes, and safeguarding cells from damage induced by various stressors [26]. Among these, HSP70 and HSP72 are known molecular chaperones that are upregulated in response to heat shock to prevent protein misfolding and cellular apoptosis [27]. The expression of HSP70 is modulated by various physiological variables, including intracellular pH, cyclic adenosine monophosphate, protein kinase C, and intracellular free calcium. Moreover, polymorphisms in the surrounding and promoter regions of the HSP70 gene have been associated with phenotypic characteristics such as heat tolerance, weaning weight, milk production, fertility, and disease resistance in animals [28].

It is well recognized that research in reproductive physiology, developmental biology, and embryology requires a sufficient number of embryos and a large population of animals. Inevitably, the sacrifice of animals comes at the cost of invaluable life, in addition to the substantial research funds needed to maintain animal populations for experimentation. To circumvent these issues, an in vitro model using mEBs generated from mouse embryonic stem cells (mESCs) has proven useful for the study of early embryogenesis [29]. Mammalian embryonic development in vivo, particularly during the post-implantation stages, presents considerable experimental challenges. This inherent inaccessibility has spurred the development of various in vitro models, including embryoid bodies, which are in vitro-derived spherical-shaped, embryo-like structures that resemble real developing embryos in forming the three germ layers. They could recapitulate some key developmental events outside the maternal environment [30]. These three-dimensional cell clusters, which develop germ layer cell lineages (ectodermal, mesodermal, and endodermal), are expected to closely mimic early developmental processes. Therefore, in the present study, we aimed to establish an in vitro model system using mEBs to investigate the effects of heat shock on peri- and post-implantation embryo development. By comparing the gene expression profiles of mEBs with *in vivo*-derived embryos under various heat shock conditions, we sought to provide a deeper understanding of how elevated temperatures affect early embryonic development and cellular stress responses.

## 2. Materials and Methods

### 2.1. Use and Care of Animals

The use and care of animals for harvesting mouse embryos strictly complied with the regulations of the Institutional Animal Care and Use Committee (IACUC) of National Chung Hsing University, Taiwan (Approval No. 103-121). Briefly, the mice were housed in cages, provided with pellet feed, and given free access to clean water. The animal room was equipped with an automatically controlled light-dark cycle, with 12 h of light and 12 h of darkness. The temperature was consistently maintained between 20 °C and 25 °C in a well-ventilated animal facility.

### 2.2. Preparation of Feeder Cells

To derive mouse embryonic fibroblasts (MEFs) as feeder cells, embryos at 13.5 days (E13.5) post-coitum were collected from the uteri of pregnant ICR female mice. The growth of cells was examined the following day, and MEFs were further cultured until reaching confluency. In this study, only MEF feeder cells at four to six passages were used [31].

### 2.3. Culture of mESCs and mEBs

Mouse ESCs were maintained in D-MEM/F-12 (Cat. No. 12400-024; Gibco Products International, Waltham, MA, USA) supplemented with 15% FBS (Cat. No. 10437-028; Gibco Products International), 2 mM L-glutamine (Cat. No. G8540-25; Sigma-Aldrich, St. Louis, MO, USA), 1% non-essential amino acids (Cat. No. M7145; Sigma-Aldrich), 0.1 mM β-mercaptoethanol (Cat. No. M7522; Sigma-Aldrich), and 1000 U/mL recombinant mouse leukaemia inhibitory factor (Cat. No. ESG1107; Chemicon, Tokyo, Japan).

### 2.4. Formation of mEBs

Mouse ESCs were digested with dispase (1 mg/mL; Gibco 17105041) at 37 °C for 2 to 3 min, gently split into small clumps, and then cultured in suspension for 1 day in the culture medium consisting of DMEM and 10% FBS. The medium was changed every other day until mEBs being later recovered for heat shock treatments and histological examination.

### 2.5. PCR Analysis

The kit used in this study (Geneaid RT050, New Taipei City, Taiwan) to extract RNA from whole mESC lines was reverse-transcribed to generate first-strand cDNA. Twenty μg of total RNA were used, to which 2 μL of DNase I, 3 μL DNase I 10X buffer, 0.25 μL of RNasin, and 4.75 μL of ddH_2_O were added for a reaction lasting 25 min at 37 °C. Primer sequences, including sense and antisense strands, annealing temperatures used in the PCR reactions, and expected product sizes, are listed in Table 1. After ethidium bromide staining, 4–10 μL of the PCR product was separated on a 1.5% agarose gel and examined under UV light. Band intensities corresponding to the PCR products were quantified by selecting the regions of interest for each band. Intensity values were corrected for background noise, and data were normalized relative to the expression of the housekeeping gene. Analysis using ImageJ (v. 1.54 g Java 1.8.0, National Institutes of Health, Bethesda, MD, USA) further substantiated these findings, confirming the enhanced expression of the target gene in the experimental group [31].

### 2.6. Real-Time PCR Analysis

The expression levels of target genes in the pituitary gland and tests were quantified by real-time PCR (qPCR). Reactions were performed using Fast SYBR™ Green Master Mix (Applied Biosystems™, Cat. No. 4385612, Waltham, MA, USA) on a StepOnePlus™ Real-Time PCR System (Thermo Fisher Scientific, Cat. No. 4376600, Waltham, MA, USA). Each 10 µL reaction contained 5.0 µL of 2× SYBR™ Green Master Mix, 3.8 µL of nuclease-free water, 0.1 µL each of forward and reverse primers (10 µM), and 1.0 µL of diluted cDNA template (1:10). The amplification program was as follows: initial denaturation at 95 °C for 5 min, followed by 40 cycles of 95 °C for 5 s and 60 °C for 30 s. A melting curve analysis was performed to verify the specificity of the amplification (95 °C for 15 s, 60 °C for 1 min, and 95 °C for 15 s). All reactions were conducted in triplicate. Gene expression levels were normalized to the expression of GAPDH, and relative expression was calculated using the 2^−ΔΔCt^ method. The expression profiles of HSP70, HSP72 and Caspase 9 gene were analyzed by real-time PCR (Table 2).

### 2.7. Immunocytochemical Staining for Specific Germ Layer Markers

To analyze the expression of specific protein markers, mEBs were rinsed with DPBS and then fixed in 4% paraformaldehyde. After washing with DPBS for 10 min, the cell membranes of mEBs were permeabilized using Tris-buffered saline with Tween-20 (TBST containing 20 mM Tris-HCl, pH 7.4, 0.15 M NaCl, and 0.05% Tween-20), treated with 0.3% Triton X-100 in DPBS for 30 min, and washed three times in DPBS before incubation with blocking solution (2% skimmed milk in DPBS) for 60 min. For staining, mEBs were first incubated with primary antibodies (anti-Pax6, Millipore MAB5552, Burlington, MA, USA; anti-Nestin, Millipore AB5922; anti-brachyury, Abcam ab20680, Cambridge, UK; and anti-AFP, Abcam ab3969) diluted in the blocking solution (Pax6: 1:250; Nestin and Brachyury: 1:200; AFP: 1:100) at 4 °C overnight. After an additional three washes (15 min per wash) with 0.05% Tween-20 (Amersham Life Science 20605, Oakville, ON, Canada), mEBs were washed again three times with DPBST and then incubated with secondary antibodies (brachyury: FITC-conjugated goat anti-rabbit IgG, 1:200, Sigma F9887; Pax6, nestin, and AFP: FITC-conjugated goat anti-mouse IgM, 1:200, Sigma F9259, St. Louis, MO, USA) for 60 min. Finally, DAPI (1 μg/mL in DPBST) was used for nuclear staining, followed by two rinses with DPBST [31].

### 2.8. Detection of Apoptosis by TUNEL Assay

A TUNEL assay was used to detect apoptotic signals in mEBs in response to thermal stress. Briefly, all mESCs and mEBs were washed twice in DPBS containing polyvinylpyrrolidone (PVP, 1 mg/mL) and then fixed in 4% (*w*/*v*) paraformaldehyde (P6148) in DPBS/PVP for 1 to 2 h at room temperature immediately following treatment. After fixation, cells were washed twice in DPBS/PVP and then permeabilised for 1 h in DPBS with 0.1% (*v*/*v*) Triton X-100. Following permeabilisation, mESCs were washed twice with DPBS/PVP and then incubated in 10 μL of terminal deoxynucleotidyl transferase and 90 μL of fluorescein-conjugated dUTP for 60 min at 37 °C in the dark. The nuclear material of cells was counterstained with propidium iodide (10 μg/mL) and Hoechst 33342 (10 μg/mL) before mounting on a slide. The slides were stored at 4 °C until examination under an epifluorescence microscope (Olympus AF-70, Tokyo, Japan).

### 2.9. Live–Dead Cell (Viability) Staining of mEBs

Mouse EBs were collected into a 15 mL centrifuge tube, the supernatant was removed, and DPBS was added for cleaning at 3000 rpm. The mEBs were then transferred to a 1.5 mL centrifuge tube, into which 100–150 μL of staining reagent from the LIVE/DEAD^®^ Viability/Cytotoxicity Assay Kit (Thermo Fisher Scientific, Waltham, MA, USA) was added to identify dead cells. The staining process lasted for 30–40 min at room temperature. To prepare the working solution, 20 µL of the supplied 2 mM EthD-1 stock solution (Component B) was added to 10 mL of sterile, tissue culture-grade DPBS, followed by vortexing to ensure thorough mixing. This resulted in an approximately 4 µM EthD-1 solution. A 5 µL aliquot of the supplied 4 mM calcein AM solution in DMSO (Component A) was then added to the 10 mL of 4 µM EthD-1 solution. The resulting solution was vortexed or sonicated to ensure thorough mixing, yielding a working solution containing approximately 2 µM calcein AM and 4 µM EthD-1. For staining, 100 µL of the cell-containing buffer was distributed into each well, followed by the addition of 100 µL of the LIVE/DEAD^®^ (Thermo Fisher Scientific, Waltham, MA, USA) working solution, resulting in a final volume of 200 µL per well. This provided final concentrations of 1 µM calcein AM and 2 µM EthD-1. The final concentration of DMSO was ≤0.1%, a level generally innocuous to most cells. For observation, the mEBs were resuspended in a 30 mm Petri dish and examined under a fluorescence microscope. Differential excitation wavelengths were used to distinguish live cells (488 nm) from dead cells (543 nm).

### 2.10. Experimental Designs

#### 2.10.1. Experiment 1: Germ Layer Gene Expression Profile

Mouse embryos were collected from the uteri of 5.5- to 8.5-day-old pregnant female ICR mice, from which total RNA was extracted for PCR analysis.

#### 2.10.2. Experiment 2: Effects of Heat Shock on Germ Layer Gene Expression in mEBs

These mEBs were randomly allocated to one of the following treatments for 12 or 24 h: the control group (37 °C) or a heat shock group (either 39 °C or 41 °C). Following treatment, the mEBs were subjected to TUNEL labelling and Live/Dead™ staining to determine apoptotic and live cells, respectively.

#### 2.10.3. Experiment 3: Effects of Various Heat Shock Regimens on HSP70, HSP72 and Caspase 9 Gene Expression

Mouse EBs were randomly allocated to one of the following treatment groups: heat shock at either a mild (39 °C) or severe (41 °C) temperature.

### 2.11. Statistical Analysis

All data from real-time PCR and immunocytochemical staining were analyzed by ANOVA using the General Linear Model (GLM) procedure in the Statistical Analysis System [32], followed by Tukey’s test. Percentile data were analyzed by completely randomized designs. For all statistical analyses, significance level was set at *p* < 0.05.

## 3. Results

### 3.1. Experiment 1: Germ Layer Gene Expression in Mouse Embryos and mEBs

**Figure 1:**
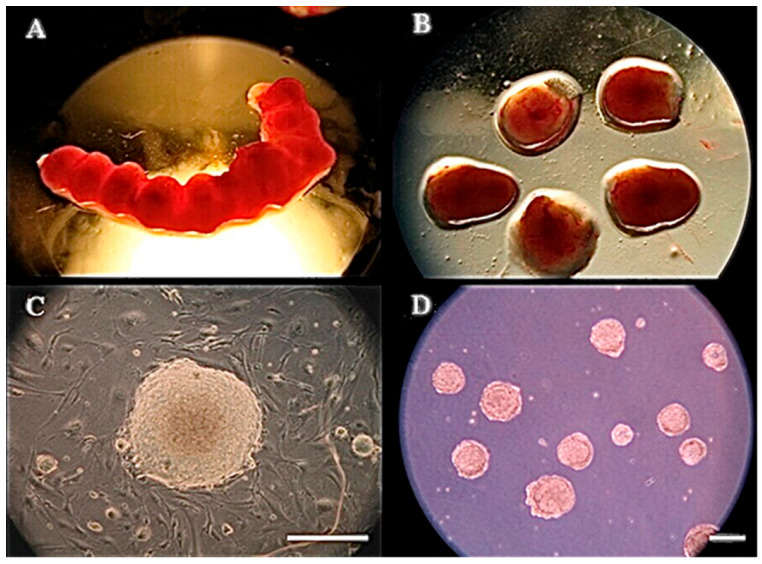
Harvested mouse embryos and embryoid bodies (EBs). (**A**) E7.5 mouse embryos developing in the uterus are dissected from the female mouse. (**B**) The uterus is dissected and cut open to release embryos enclosed in the fetal membranes. (**C**) Morphology of a mouse ES (mES) cell colony cultured on the feeders and then (**D**) cultured in suspension to form EBs for gene and protein analyses. Scale bar = 200 μm.

**Figure 2:**
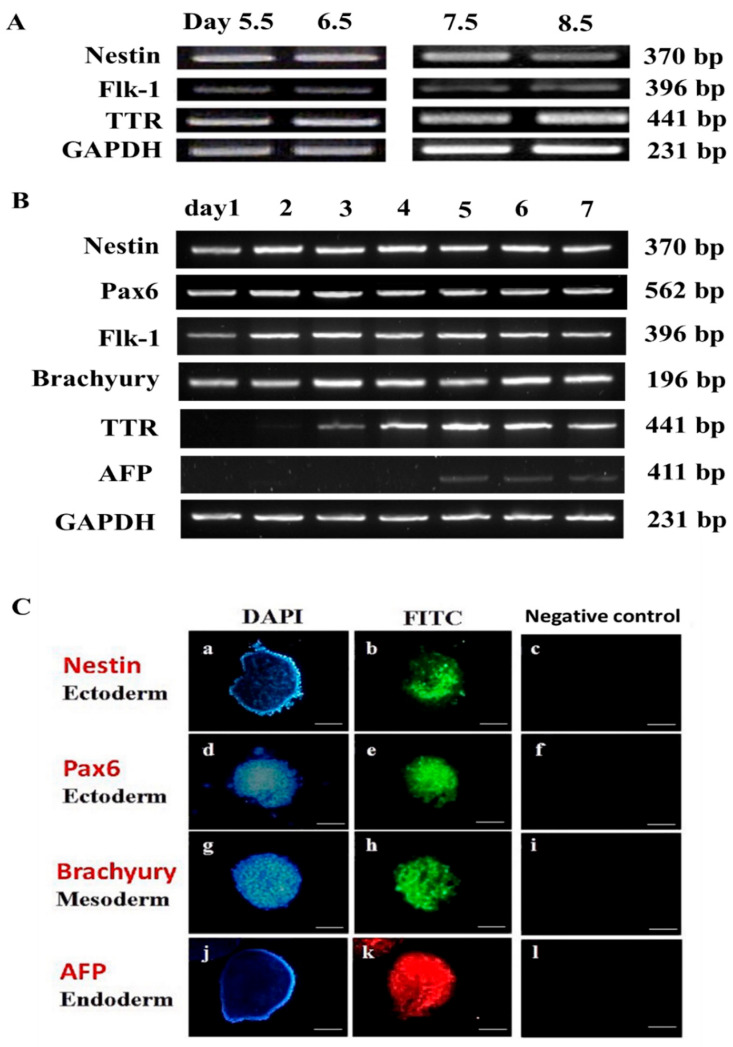
Marker gene expressions of the three germ layers detected by PCR and Immunohistochemistry. (**A**) E5.5–E8.5 mouse embryos were used for each experimental group (*n* = 30); (**B**) Days 1–7 mEBs (*n* = 350). (**C**) Mouse EBs express ectodermal protein markers nestin (**b**) and pax6 (**e**), mesodermal marker brachyury (**h**) on Day 4, and endodermal markers afp (**k**) on Day 7. DNA was counterstained with DAPI (**a**,**d**,**g**,**j**). No 1st antibody: (**c**,**f**,**i**,**l**). Scale bar = 200 μm. Marker genes: ectoderm, *nestin*; *mesoderm*, *flk-1*; endoderm, *ttr*. Control, *gapdh*.

The gene expression profile of the three germ layers in mouse embryos harvested from the uterus between 5.5 and 8.5 days old (E5.5–E8.5) (Figure 1A,B) was compared with that of 1- to 7-day-old mEBs (Figure 1C,D). The results showed that E5.5 embryos expressed three germ layer marker genes: *nestin* (ectoderm), *flk-1* (mesoderm), and transthyretin (*ttr*; endoderm) (Figure 2A). At E6.5, marker genes including *nestin*, *flk-1*, and *ttr* were expressed. During E7.5–E8.5, however, embryos continuously expressed all key genes of the three germ layers (Figure 2A).

Similarly, newly formed mEBs (designated as EB day 1 [EBD1]) began expressing most of the ectodermal marker genes (*nestin* and *pax6*) and the mesodermal markers *flk-1* and *brachyury*, but not the endodermal markers *ttr* and *afp*. The *ttr* gene appeared to be first detected at a trace level on EBD2 and became more prominently expressed on EBD3, while *afp* only became detectable starting from EBD5 in culture (Figure 2B). When comparing mEBs with in vivo-derived embryos, the gene expression profile of EBD1 exhibited partial similarity to that of E5.5 embryos, with ectodermal (*nestin*) and mesodermal (*flk-1*) markers expressed, while endodermal (*ttr*) expression had not yet been detectable. This aligns with the fact that EBD1 cells were derived from the inner cell mass of blastocysts at day 4.5, with an additional 1-day culture period for EB formation. EBD1 expressed the ectodermal marker *nestin* and the mesodermal marker *flk-1* (Figure 2B), but not the endodermal marker *ttr*. This finding delineates the gene expression profile differences between mEBs and corresponding embryos (Figure 2A). All marker genes were expressed at EBD2, EBD3, and EBD5 except for AFP expression only being detectable starting from EBD5. As shown in Figure 2C, major germ layer marker proteins, such as nestin (Figure 2C(b)) and brachyury (Figure 2C(h)) on Day 4, and afp (Figure 2C(k)) on Day 7, were observed by immunocytochemical staining.

### 3.2. Experiment 2: Expression of Germ Layer Marker Genes in Heat-Shocked mEBs

**Figure 3:**
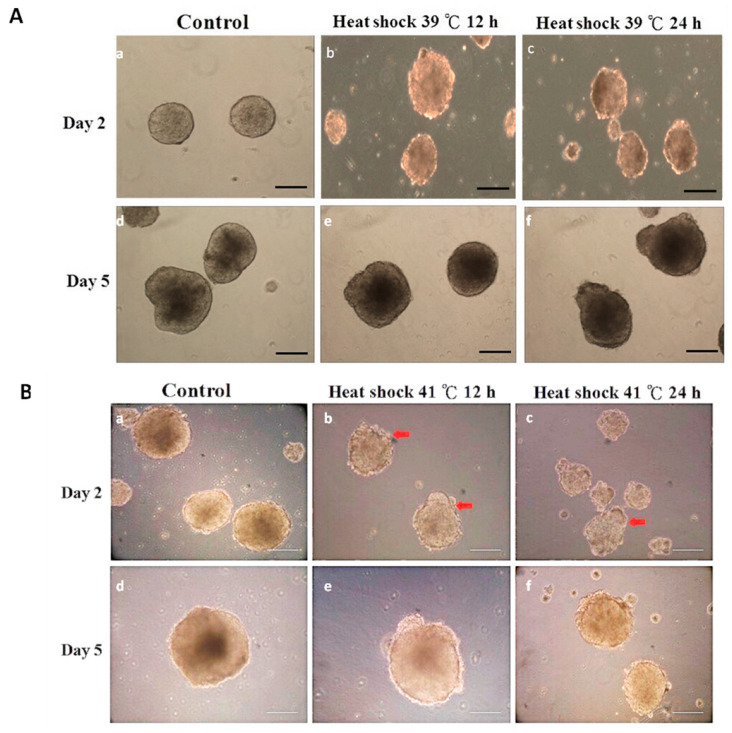
Morphological alterations of Day 2 and Day 5 EBs after heat-shock (HS) treatment at 39 °C (**A**) and 41 °C (**B**) for 12 h or 24 h, respectively. (**A**) EBs of the control group (37 °C, (**a**,**d**)) show a smooth and defined boundary; however, the EBs after HS at 39 °C for 12 h (**b**,**e**) and HS at 39 °C for 24 h (**c**,**f**) have a relatively rough boundary compared with those of the control group. (**B**) EBs of the control group (37 °C, (**a**,**d**)) show a smooth and defined boundary; when the EBs were heat-shocked at 41 °C for 12 h (**b**,**e**) and 41 °C for 24 h (**c**,**f**), morphological deterioration was observed on both Day 2 and Day 5 EBs. The arrows were included to indicate specific regions of alterations by heat shock. Scale bar = 200 μm.

**Figure 4:**
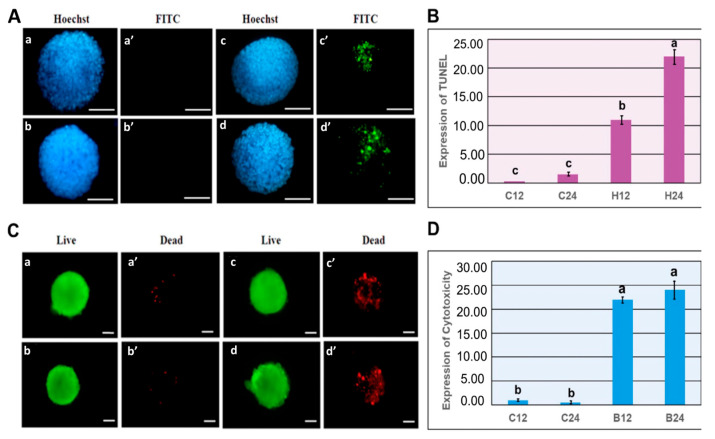
Apoptosis and live-dead cell ratios of EBs detected by TUNEL and viability assay kits under heat-shock conditions (41 °C for 12 h or 24 h). (**A**) EBs of the control (37 °C) group were cultured for 12 h (**a**,**a’**) and 24 h (**b**,**b’**) with no apoptotic cells observed. In the heat shock group (41 °C) for 12 h (**c**,**c’**) and 24 h (**d**,**d’**), clear apoptotic signals were shown. DAPI staining (**a**–**d**) and apoptotic nuclei (green, FITC); (**a’**–**d’**) are labeling TUNEL staining. (**B**) The quantitative TUNEL signals were examined and summarized. Scale bar = 100 μm. (a–c): *p* < 0.05. (**C**) Live and dead cells of EBs detected by using LIVE/DEAD Viability/Cytotoxicity Assay Kit under heat-shock conditions (HS at 41 °C for 12 h or 24 h). In the control group (37 °C), the EBs were cultured for 12 h (**a**,**a’**) or 24 h (**b**,**b’**). In the heat-shocked groups, the EBs were heated at 41 °C for 12 h (**c**,**c’**) or 24 h (**d**,**d’**). (**a**–**d**) Representative micrographs of live cells (green) and (**a’**–**d’**) dead cells (red). (**D**) The quantitative LIVE/DEAD Viability/Cytotoxicity Assay Kit ratios were summarized and compared. Scale bar = 50 μm. (a, b): *p* < 0.05.

**Figure 5:**
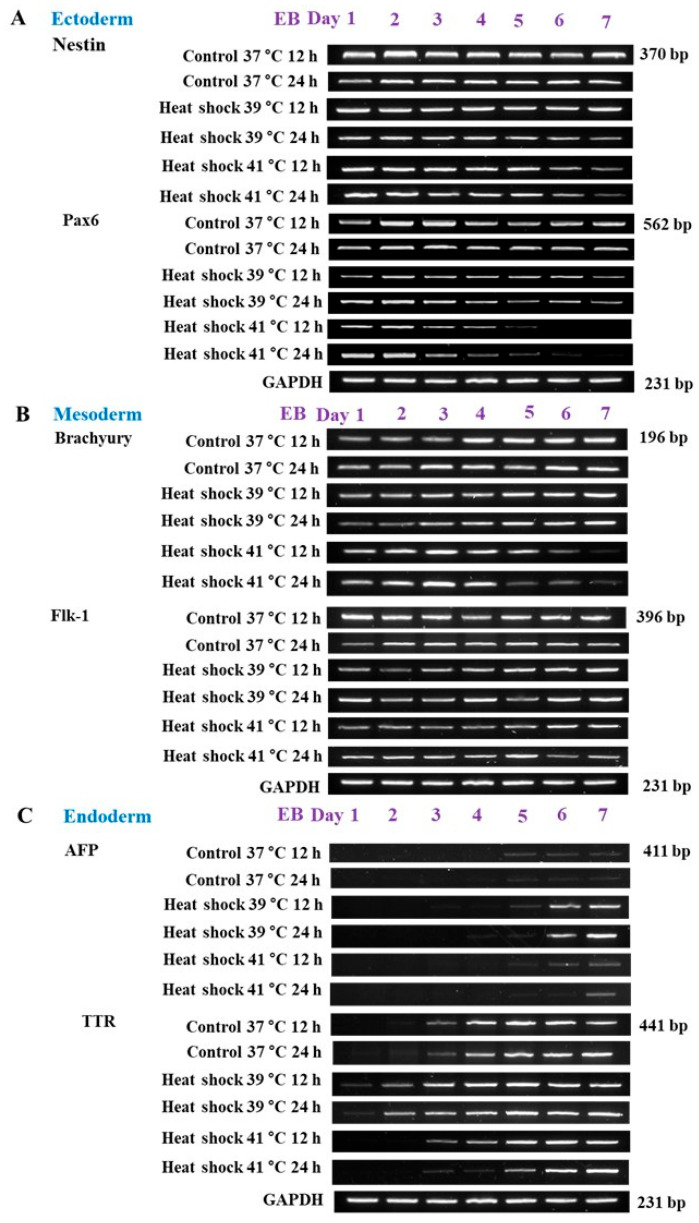
Analysis of the expression of three marker genes at Days 1 to 7 for mEBs after heat shock treatments. (**A**) *nestin* and *pax6* Ectodermal gene, (**B**) *afp* and *ttr* endodermal and (**C**) *brachyury* and *flk-1* mesodermal marker gene expressions analyzed by PCR. Days 1 to 7 mEBs were randomly allocated to five treatment groups, i.e., 12 or 24 h (control, 37 °C), mild heat shock (39 °C) for 12 h or 24 h, and severe heat shock at 41 °C for 12 h or 24 h. gapdh: internal control; 39 °C and 41 °C: heat shock groups.

**Figure 6:**
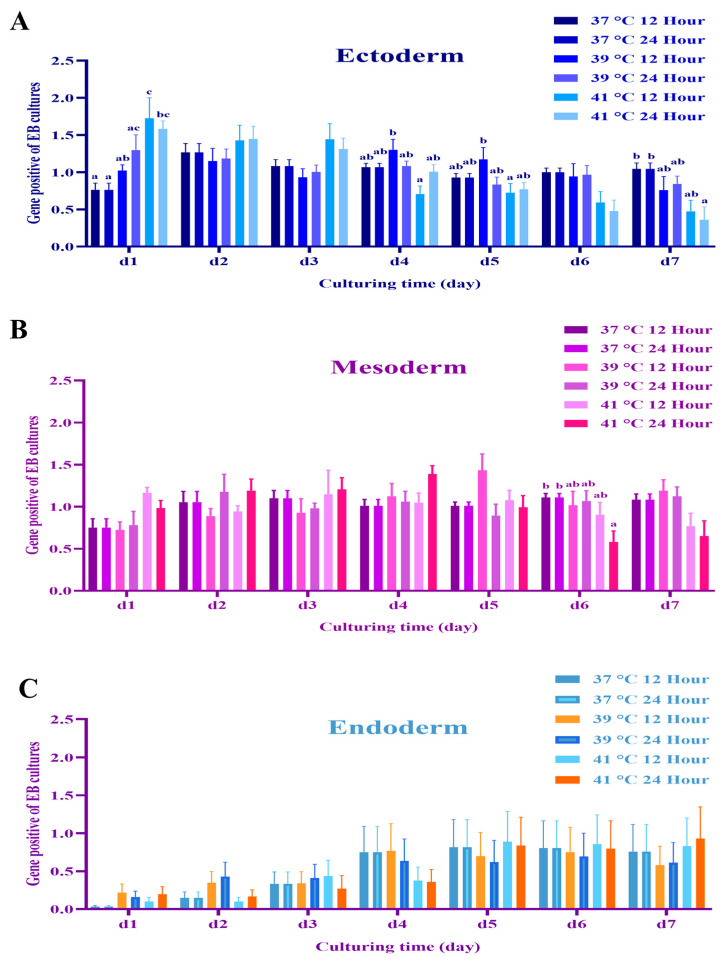
The relative expression levels of the PCR analyses of the markers shown above on 1 to 7 days of the germ layer. Control groups for 12 or 24 h (37 °C), and heat shock groups for 12 or 24 h (39 °C or 41 °C). (**A**) Ectoderm (*nestin* and *pax6*) for 12 or 24 h. (**B**) Mesoderm (*brachyury* and *flk-1*) for 12 or 24 h. (**C**) Endoderm (*afp* and *ttr*) for 12 or 24 h. The error bars indicate standard deviation and data presented as mean ± SEM of three independent experiments. The difference was considered significant when a, b, c: *p* < 0.05. n = 3.

Mouse EBs were randomly allocated to experimental groups. The control mEBs (under 37 °C) displayed smooth and uniform contours with clearly defined peripheral organization (Figure 3A). In contrast, when exposure to heat stress at 41 °C for 12 h and 24 h, mEBs underwent pronounced structural degradation. Morphological deterioration in structural integrity or cellular disorganization was evident in both Day 2 and Day 5 embryoid bodies subjected to the elevated temperature (Figure 3B). The mEBs were randomly allocated to one of the following treatments control conditions at 37 °C for 12 and 24 h exhibited minimal TUNEL reactivity, indicating the absence of notable apoptotic activity. In contrast, EBs subjected to heat shock at 41 °C for 12 and 24 h demonstrated a marked increase in TUNEL-positive nuclei, visualized as green fluorescence (FITC), indicative of extensive DNA fragmentation associated with apoptosis (Figure 4A). Quantitative analysis of apoptosis using TUNEL signal detection revealed a statistically significant increase (*p* < 0.05) in the proportion of apoptotic cells within EBs subjected to heat shock at 41 °C for both 12 and 24 h, when compared with the corresponding time-matched control groups (Figure 4B). The viability/cytotoxicity assay revealed that control cultivated at 37 °C for 12 and 24 h displayed primarily green fluorescence, elevated as cell viability. Conversely, EBs exposed to heat shock at 41 °C for 12 and 24 h exhibited significant red fluorescence indicative of EthD-1 uptake, signifying a large rise in non-viable cells (Figure 4C). The quantitative evaluation of cell viability demonstrated a notable reduction in the percentage of viable cells, and a concomitant rise in cytotoxicity. The embryoid bodies exposed to heat shock at 41 °C for both 12 h and 24 h exhibited a significantly higher rate of cell death compared to the control groups (*p* < 0.05) as illustrated in Figure 4D.The ectodermal markers nestin and Pax6 exhibited strong expression at Day 1 across all groups; however, with extended or intense heat stress (notably 41 °C for 24 h), expression levels decreased in subsequent stages (Days 4–7) in Figure 5A. The expression of nestin and Pax6 was considerably increased (*p* < 0.05) on Day 1 in the 41 °C (for 12 h and 24 h) groups compared to the control, demonstrating an acute stress-induced up-regulation of ectodermal transcription. As differentiation advanced, the expression levels of these markers dramatically decreased during Days 4–7 in those groups subjected to 39 °C for 12 h and 41 °C for 24 h (*p* < 0.05), indicating persistent down-regulation of ectodermal lineage commitment (Figure 6A). A comparable trend was noted for the mesodermal markers Brachyury and flk-1, whose expressions were consistently diminished in the 41 °C for 24 h group compared to the controls (Figure 5B). Likewise, Brachyury and flk-1 expressions significantly diminished during culture in the 41 °C for 24 h group (*p* < 0.05), corroborating the inhibitory impact of high-temperature stress on mesodermal development. This data indicates that mesodermal differentiation is particularly susceptible to prolonged heat exposure (Figure 6B), while the endodermal markers displayed different responses. In TTR expression, it had a delayed expression beginning in the most severely heat-shocked group (41 °C for 24 h), manifesting about one to two days later than in the control (Figure 5C). AFP expression, however, remained subdued across all treatments, complicating the detection of potential delays due to its low baseline level in the control mEBs (Figure 6C). The results collectively suggest that increased temperature primarily leads to the down regulation of ectodermal and mesodermal gene expressions during mEB differentiation, while endodermal differentiation demonstrates relative resilience, exhibiting delayed TTR expression under extreme heat-shock conditions.

### 3.3. Experiment 3: Effects of Mild and Severe Heat Shock on HSP70, HSP72 and Caspase-9 Expression in mEBs

Gene expression ratios of HSPs were assessed using real-time PCR at 12 and 24 h following treatment. Compared to the control group at 37 °C, the mRNA expression levels of both constitutive *HSP70* isoforms were considerably increased in mEBs exposed to heat shock at 39 °C or 41 °C, with the most marked upregulation observed at the 24 h point.

**Figure 7:**
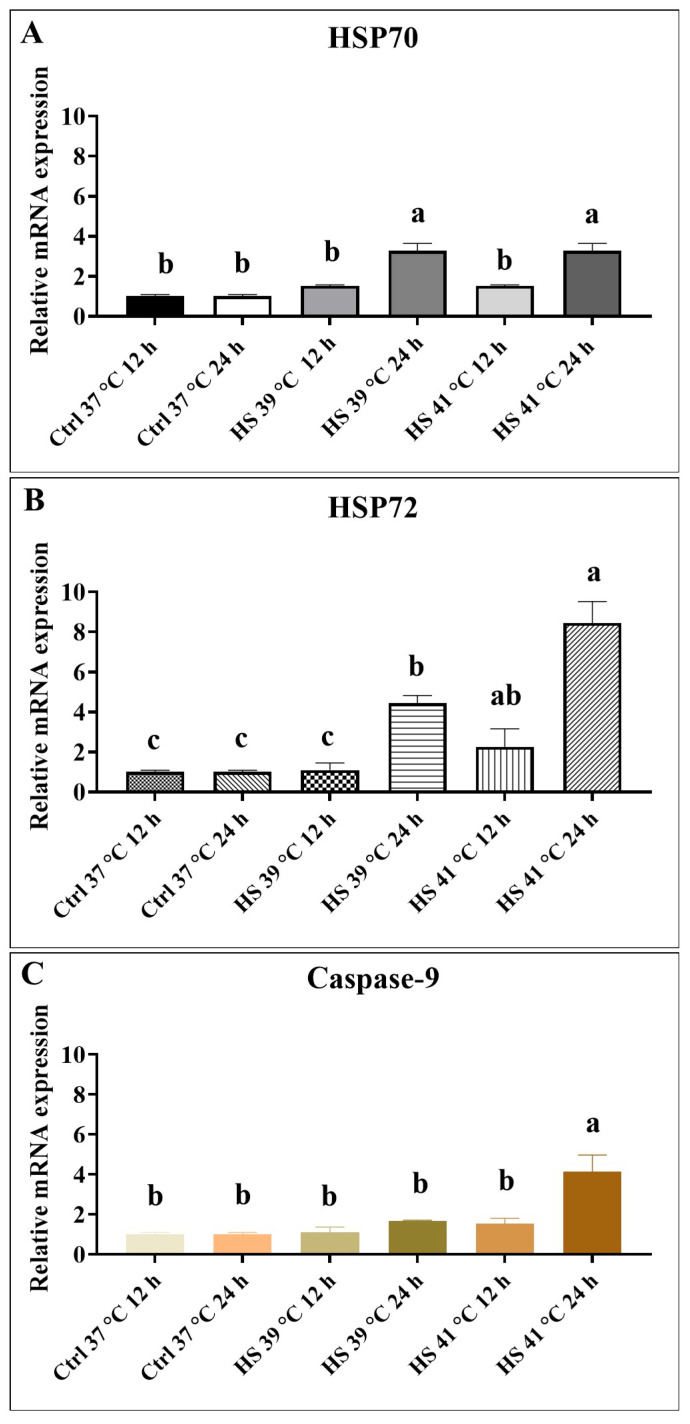
Relative mRNA expression levels of the real time PCR analyses of mouse embryoid bodies derived from mouse embryonic stem cells. (**A**) *HSP70*, (**B**) *HSP72*, and (**C**) Apoptosis-related gene *Caspase-9*. Mouse embryoid bodies were heat shock 39 and 41 °C for 12 and 24 h. Ctrl: Control groups non-heat shock 37 °C for 12 and 24 h. The error bars indicate standard deviation and data presented as mean ± SEM of three independent experiments were performed for each group. Bars without the same alphabetic letters differ. a, b, c: *p* < 0.05. “a” represents the highest value. Three replicates.

The expression of *HSP72* was markedly elevated in mEBs subjected to 41 °C for both 12 and 24 h in comparison to time-matched controls. A significant increase (*p* < 0.05) in *Caspase-9* mRNA levels was noted after 24 h of exposure to 41 °C heat shock, with expression significantly elevated compared to all other treatment groups (Figure 7).

## 4. Discussion

The mEB model was then utilized to investigate the effects of heat shock on germ layer gene expression in differentiating embryos during the peri- and post-implantation stages. The results offer valuable insights into how exposure to heat shock at 39 °C and 41 °C for 12 and 24 h influences the expression of genes related to the three germ layers, induces apoptosis, and regulates the levels of HSP70 and HSP72. These temperature-induced changes suggest that heat stress disrupts normal embryonic development by interfering with key molecular pathways. Heat stress is a critical environmental factor that negatively affects reproductive performance and embryonic development in livestock, particularly in tropical and subtropical regions [1]. Both mEBs and embryos exhibited essential marker genes for the three germ layers: *nestin* for the ectoderm, *flk-1* for the mesoderm, and *ttr* for the endoderm. The delayed expression of the endodermal markers *ttr* and *afp* in mEBs compared with *in vivo* embryos may be attributed to variations in the in vitro environment affecting differentiation timing. Previous studies have shown that the expression of endodermal genes in EBs during culture closely reflects the gene expression patterns observed in normal embryo development [33]. These findings confirm that mEBs serve as an appropriate in vitro model for investigating early embryonic development and germ layer differentiation, aligning with prior research highlighting the value of EBs in developmental biology. Additionally, bioengineered embryoids have been shown to mimic post-implantation development *in vitro* [30]. Three-dimensional embryoid models initiate self-organization processes that closely resemble early embryonic development, addressing the variability and asynchronous developmental patterns commonly observed in EBs [34].

Based on our findings, the mEB model system effectively simulates peri-implantation embryos, as demonstrated in Experiment 2. We clearly showed that the effects of heat shock on the gene expression patterns of mEBs closely resembled those observed in peri-implantation embryos. Previous studies have revealed that when mouse neural stem cells were exposed to heat shock at 42 °C for 20 min, their proliferative capability decreased, albeit with only slight modifications in gene expression, cell division, proliferation, and differentiation [35]. However, no studies to date have specifically examined how heat shock influences germ layer differentiation in animal cells. Therefore, our second objective was to understand how differentiating germ layers respond to environmental or physiological thermal stress. In our previous study, we reported that the viability of a matured cattle oocyte could only be sustained for 45 min of 41 °C [11,13]. Morphological analysis of mEBs exposed to heat shock revealed that cells surrounding the peripheral layer degenerated and increased apoptosis, suggesting that elevated temperatures interfere with normal embryonic development [14]. This sensitivity to heat stress aligns with studies indicate that thermal stress can hinder cell cycle progression and disrupt differentiation pathways [18].

Heat-shocked mEBs exhibited relatively higher expression of endodermal markers compared with ectodermal and mesodermal markers, indicating that endodermal differentiation may be less adversely affected by thermal stress. Ectodermal and mesodermal cells exhibit heightened sensitivity to increased temperature. In conjunction with mEB formation or differentiation media, these stressors facilitate lineage-specific differentiation through the activation of target genes, such as the hypoxia response element, antioxidant-responsive element, electrophile-responsive element, and heat shock-responsive element [36]. The current study revealed that in vitro heat shock postponed the expression of germ layer marker genes while increasing HSP72 expression in mEBs. HSPs play a crucial role in protecting cells from stress-induced damage [21].

In Experiment 3, HSP70 was consistently expressed across all conditions, with a marked increase observed under intense heat shock at 41 °C. The inducible HSP72 was significantly elevated in response to higher temperatures and prolonged heat exposure, reflecting the activation of cellular defense mechanisms. In response to cellular stress, heat shock factor 1 (HSF1) is activated and induces the transcription of genes encoding molecular chaperones, serving as a protective mechanism against cellular damage [37]. Under conditions of heat shock, oxidative stress, and proteotoxic stress, HSF1 enhances the transcription of HSPs, which function as molecular chaperones to protect cells from stress and various pathological conditions. Emerging evidence indicates that HSF1 modulates diverse forms of cell death by influencing multiple signaling pathways and regulating specific target genes [38]. Additionally, the elevated expression of caspase-3 in heat-exposed mEBs indicates increased apoptotic activity, highlighting the relationship between heat stress, HSP induction, and apoptosis. Caspase-3, a key member of a conserved protein family, plays a critical role in facilitating apoptosis through proteolytic activation in response to specific extrinsic or intrinsic death signals within cells [39]. The role of caspase-3 underscores the detrimental effects of heat stress on cellular viability and developmental capacity. Similar findings have been reported in bovine embryos, where heat stress induced apoptosis and negatively impacted fetal development [19].

## 5. Conclusions

**Figure 8:**
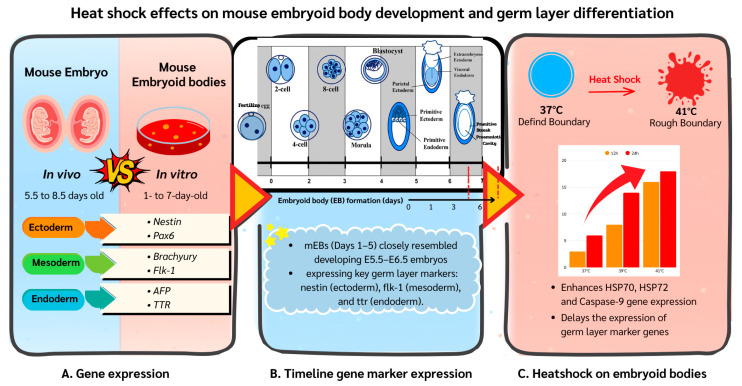
(**A**) Gene expression in 1–7-day embryoid bodies (EBs) matched with 5.5–8.5-day mouse embryos, demonstrating their model status. (**B**) Lineage specification is initiated and achieved during early-stage EBs (Days 1–5) that exhibit germ layer marker expressions (*Nestin*/ectoderm, *Flk-1*/mesoderm, *TTR*/endoderm). (**C**) Heat shock (41 °C) interrupts EB growth, creating structural damage and rough cellular borders. Stress genes (*HSP70*, *HSP72*, *Caspase-9*) are significantly upregulated in a time-dependent manner while germ layer marker expressions are delayed, indicating a defective differentiation.

Mouse EBs closely mimic *in vivo*-developing embryos in their germ layer gene expression profiles and serve as a simplified model system for studying peri-implantation embryos. In vitro heat shock delays the expression of germ layer marker genes and enhances *HSP70*, *HSP72* and *Caspase-9* gene expression in the mEB system (Figure 8). Potentially, such an mEB model system could also provide an in vitro platform for studying embryo development of other domestic species, such as rabbits, pigs, and other animals. Further refinement of this model system could also reduce the need for animal sacrifice in laboratory research.

## Figures and Tables

**Table 1:** Primer sequences and conditions of mouse genes for PCR analysis.

Gene	Primer Sequences (Forward/Reverse)	Annealing Temperature	Products Size	Accession Numbers
Nestin	F-AGCAACTGGCACACCTCAA R-ACATCCTGGGCTCTGACCT	59 °C	370 bp	NM_016701.3
Pax6	F-AGACTTTAACCAAGGGCGGT R-TAGCCAGGTTGCGAAGAACT	58 °C	562 bp	NM_013627.6
Flk-1	F-AGGTGCCTCCCCATACCCTGG R-GGCCGGCTCTTTCGCTTACTG	65 °C	396 bp	NM_010612.3
Brachyury	F-ATAACGCCAGCCCACCTAC R-CGGAGAACCAGAGACGAG	58 °C	196 bp	NM_009309.2
TTR	F-AGTCCTGGATGCTGTCCGAG R-TTCCTGAGCTGCTAACACGG	59 °C	441 bp	NM_013697.5
AFP	F-GCTCACACCAAAGCGTCAAC R-CCTGTGAACTCTGGTATCAG	54 °C	411 bp	NM_007423.4
GAPDH	F-GAGCCAAACGGGTCATCATCT R-AGGGGCCATCCACAGTCTTCT	65 °C	231 bp	NM_008084.4

**Table 2:** Primer sequences and conditions of mouse genes for real-time PCR analysis.

Gene	Primer Sequences (Forward/Reverse)	Products Size	Accession Numbers
HSP72	F-ACGGCATCTTCGAGGTGAA R-TGTTCTGGCTGATGTCCTTCT	129 bp	NM_010479.2
HSP70	F-AAGAACGCGCTCGAGTCCTAT R-GAGATGACCTCCTGGCACTTGT	122 bp	NM_010478.3
Caspase 9	F-TTGTCTCCTGGAGGGACAAGAA R-AAGGAGGGACTGCAGGTCTTC	101 bp	NM_015733.6
GAPDH	F-CTGCACCACCAACTGCTTAGC R-CAGTCTTCTGGGTGGCAGTGA	110 bp	NM_008084.4

## Data Availability

Most of the data constituting the above findings are available upon request, while some might be unavailable at this time as they also form part of an ongoing research project.
